# Clinical and Research Criteria for Developmental Coordination Disorder—Should They Be One and the Same?

**DOI:** 10.1007/s40474-015-0043-9

**Published:** 2015-03-11

**Authors:** Reint H. Geuze, Marina M. Schoemaker, Bouwien C. M. Smits-Engelsman

**Affiliations:** 1 Clinical and Developmental Neuropsychology, University of Groningen, Grote Kruisstraat 2-1, 9712 TS Groningen, The Netherlands; 2 Centre for Human Movement Sciences, University of Groningen, University Medical Centre Groningen, PO Box 30.001, 9700 RB Groningen, The Netherlands; 3 Faculty of Kinesiology and Rehabilitation Sciences, Katholieke Universiteit, Leuven, Belgium; 4 Department of Health and Rehabilitation Sciences, Faculty of Health Sciences, University of Cape Town, Old Main Building, Groote Schuur Hospital, Cape Town, South Africa

**Keywords:** Cutoff criteria, Research criteria, Motor development, Motor problems, Motor proficiency, Motor skill

## Abstract

The aim of this paper is to discuss if criteria used for diagnosing children for clinical purposes should be the same as for the selection of children with Developmental Coordination Disorder for research. Next, we give an overview of the criteria mentioned in the development of the European guideline for diagnosing Developmental Coordination Disorder and the implementation of this guideline in different countries. To gain insight into current clinical practice, we also reviewed the medical files of children attending rehabilitation centers for the criteria used to diagnose Developmental Coordination Disorder in the Netherlands. To conclude, we state our expert opinion on why and when research and clinical criteria for Developmental Coordination Disorder should or should not be the same.

## Introduction

In the 2001 special issue of Human Movement Science on Developmental Coordination Disorder (DCD), the topic of research and clinical diagnostic criteria for DCD was discussed for the first time [1] by Geuze, Jongmans, Schoemaker, and Smits-Engelsman. In 2001, we wrote: “ …the Diagnostic and Statistical Manual for Mental Disorders (DSM)-IV provides qualitative criteria for clinical purposes. As our review has shown, these criteria are met in a variety of ways and cut-off criteria for motor performance and IQ differ across studies. It is also apparent that additional criteria are frequently used. Together, these findings led us to explore the possibility of devising a protocol, separating clinical diagnostic criteria from research diagnostic criteria, from which we can develop a unifying view on these two types of criteria for research and clinical practice.” Now that DSM-5 [2] has been published and some 12 years of new publications on DCD have appeared (for a review of criteria used since 2001, see [3]), we feel it is appropriate to reconsider the distinction between clinical and research diagnostic criteria. Since the DSM is the leading system to classify DCD in clinical practice and research, we build upon its set of criteria. Table 1 lists the diagnostic criteria for DCD of DSM-IV and DSM-5.

**Table 1:** DSM-IV [4] and DSM-5 [3] diagnostic criteria for Developmental Coordination Disorder

DSM-IV criteria	DSM-5 criteria
A. Performance in daily activities that require motor coordination is substantially below that expected given the person’s chronological age and measured intelligence. This may be manifested by marked delays in achieving motor milestones (e.g., walking, crawling, and sitting), dropping things, “clumsiness”, poor performance in sports, or poor handwriting).	A. The acquisition and execution of coordinated motor skills is substantially below that expected given the individual’s chronological age and opportunity for skill learning and use. Difficulties are manifested as clumsiness (e.g., dropping or bumping into objects) as well as slowness and inaccuracy of performance of motor skills (e.g., catching an object, using scissors or cutlery, handwriting, riding a bike, or participating in sports).
B. The disturbance in Criterion A significantly interferes with academic achievement or activities of daily living.	B. The motor skills deficit in Criterion A significantly and persistently interferes with activities of daily living appropriate to chronological age (e.g., self-care and self-maintenance) and impacts academic/school productivity, prevocational and vocational activities, leisure, and play.
C. The disturbance is not due to a general medical condition (e.g., cerebral palsy, hemiplegia, or muscular dystrophy) and does not meet criteria for a Pervasive Developmental Disorder.	C. Onset of symptoms is in the early developmental period.
D. If Mental Retardation is present, the motor difficulties are in excess of those usually associated with it.	D. The motor skills deficits are not better explained by intellectual disability (intellectual developmental disorder) or visual impairment and are not attributable to a neurological condition affecting movement (e.g., cerebral palsy, muscular dystrophy, degenerative disorder).

## Development of a European Guideline for DCD and the Implementation of the Guideline

Using the same standard for diagnosis of DCD across communities and countries will help determine the prevalence of DCD and how health and education systems are affected by this condition. In 2004 and 2005, four consensus meetings were held in Leeds (UK), where a group of international clinicians and researchers aimed to establish the operational definition of the diagnostic criteria for DCD. The results of these consensus meetings were published in the Leeds consensus statement [5].

Parallel to this initiative, the Dutch DCD network consisting of a steering committee and three groups of professionals working with children with DCD held a similar series of meetings in the Netherlands, to reach consensus about the operational definition of the DSM-IV [4] and later on the DSM-5 criteria [3] for use in clinical practice. The process to reach consensus followed an empirical circle: a research question was formulated and investigated in clinical practice, after which the results were discussed by the steering committee. Next, the steering committee proposed an operational definition for the diagnostic criteria, which was first discussed in the professional working groups. Then, a definite proposal was formulated and presented at a national policy conference to reach consensus about the proposal. This cyclical process led to consensus about the operational definition of the diagnostic criteria for DCD for use in the Netherlands in 2008.

Around 2008, Rainer Blank, on behalf of the European Academy of Childhood Disability (EACD), initiated the development of an international consensus statement. A group of international experts on DCD collaborated on the development of an evidence-based guideline for the definition, assessment, and intervention of DCD. After an extensive literature search, several recommendations were formulated, which were discussed following the Delphi Round system to reach consensus and were approved by an international panel of experts in 2010. Some of these experts were previously involved in the Leeds and Dutch consensus meetings. This process resulted in the publication of the international EACD recommendations for definition, diagnosis, and assessment [6]. This comprehensive consensus document provides the evidence-based recommendations for unified terms of reference in DCD. Consequently, in the Netherlands, the UK, and the German-speaking countries, workshops or consensus meetings were held to discuss the feasibility of the international recommendations regarding the operational definition of DCD. At the conclusion of these workshops, the international recommendations were accepted with minor revisions to fit in the country-specific context adapted for use in clinical practice (Germany [6]; UK [7]).

## Implementation of DCD Diagnostic Criteria in the Netherlands: a Review Medical Files

In order to assess the feasibility of the Dutch operational definitions of the DSM-IV criteria (which are in line with the international recommendations), we conducted a retrospective study. The medical records of a sample of 107 children between 5 to 12 years, referred to rehabilitation clinics in the Netherlands for motor skill deficits in the years 2006 and 2007, were screened to determine adherence to the four DSM-IV diagnostic criteria for DCD. Of these 107 children, 86 (80 %) met all four criteria. Twenty-one cases (20%) did not meet criterion A, as performance on the MABC was not below the 15th percentile. In order to determine the clinical status of these children, who did not meet criterion A despite being referred for motor problems at home or at school (criterion B), the medical records of these 21 children were further examined. Two of these 21 children also appeared not to meet criterion D, one due to a neurological impairment and one due to an IQ below 70 (children with IQ scores below 70 are not classified as having DCD according to the Dutch operational definition of the DSM-5 criteria). Closer inspection of the medical records of the remaining 19 children revealed that 13 of these children were primarily referred for problems with handwriting. This means that these children were correctly classified in the non-DCD group, as children with only handwriting problems without fine motor problems cannot be classified as DCD. Only six out of the 107 children (6 %) failed to meet other diagnostic criteria for DCD, although they had been referred for motor skill deficits; in other words, these children should be considered as false positives. Thus, we may conclude that the current operational definition of the diagnostic criteria in the Netherlands captures the group of children referred for motor problems to rehabilitation center DCD quite well.

## Should Clinical and Research Criteria for DCD Be the Same?

Clinical and research diagnostic criteria serve different purposes. Evidently, clinical criteria should be used to diagnose referred cases and to confirm suspected cases arising from screening tests. In these instances, proper diagnosis and labeling is a requirement for decisions related to access to special education, treatment, remedial teaching, and reimbursement of costs related to services rendered by medical insurance schemes. The use of research diagnostic criteria, in contrast, ensures the selection of a sample that is optimally suited for answering the research question. This is true independent of the source from which children are selected, the main sources being clinical referrals and screening procedures.

The criteria for a diagnosis have been laid down in the DSM-5 manual (see Table 1), and guidelines have been established to support and unify the practical application of the criteria in diagnostic assessment. Considering the research published over the last 12 years, we may conclude that the four recommendations put forward in our 2001 paper [1, pp 30–34] remain relevant today (notably, this paper has been cited over a hundred times). In many papers, however, the number of children is still small and the DCD group ill-defined (i.e., including children with poor motor performance scores with a very limited or no report of problems pertaining to activities of daily living or academic achievement). This severely hampers conclusions related to understanding the condition of DCD. Therefore, we add some further guidance below.

Research diagnostic criteria should be carefully tuned to the research questions addressed. Research into the etiology of, the factors related to, and the consequences for activities of daily living of DCD requires strict labeling and diagnosis, but may also require a wide range of children not fully meeting the criteria to answer certain questions. For example, clinical questions require well-defined clinical groups, as in: “What is the prevalence of ADHD in children (diagnosed) with DCD?” and “Do children with DCD benefit from intervention?”. Here, the inclusion and exclusion criteria should be identical to the clinical diagnostic criteria.

However, questions that essentially address a relationship such as “Do balance problems relate to motor proficiency in children with DCD? require a different approach. One should realize that the measures of motor skill and quality of ADL in the population are performance measures on a continuum. Therefore, one needs to take a perspective beyond the domain of DCD, because this question requires a wider investigation of the relationship between balance control and motor proficiency in the general population (see further discussion below). Moreover, such relationships may be task dependent and dynamic, that is, may change over time—developmental time or the time scale of intervention. The groups involved in this type of research should be defined by inclusion and exclusion criteria specified by the research question rather than pure clinical diagnostic criteria.

To further illustrate our position, we clarify the use of research diagnostic criteria further using the following five research questions as examples:

1. Do children with DCD differ from typically developing (TD) children on X? (X being some characteristic hypothesized to be different between groups). This is an exploratory question; its answer would specify the characteristics of children with DCD on a descriptive level.
2. Is motor performance related to IQ in children with DCD? This is a research question without any causal assumption; the relationship may be due to interaction between motor and cognitive capacities during development.
3. Does Y explain the poor motor performance of children with DCD? (Y being a functional measure such as attentional capacities, gestational age, lack of experience, poor socioeconomic conditions). This question assumes a causal relationship (but does not prove it without manipulation of Y).
4. Do the characteristics of children with DCD differ from other clinical groups? This should answer the question of specificity and sensitivity of relevant measures.
5. Do children with DCD have an internal modeling deficit? This is a question with a theory-based hypothesis. The answer adds to a theoretical explanation of a (specific) deficit in children with DCD.

As questions 1, 4, and 5 specifically address children with DCD, the full set of clinical criteria for the diagnosis of DCD should be used to select the children for the DCD group. The TD group should have none of the signs specified in the clinical criteria for DCD and a motor performance score above the 15th percentile.^1^

Questions 2 and 3 also refer to children with DCD, but there is an implicit reason to take a wider perspective that includes the borderline and the normal range of motor proficiency. For question 2, the motor performance range may be too small (i.e., <15th percentile) to show any significant relationship if studied within the DCD group alone. A better approach is to study this relationship over a wide range of motor performance and IQ and compare the distributions of the IQ data and motor performance data between children with and without DCD [8]. Linear and/or quadratic regression analyses over and within groups may provide a robust answer to the question. As to question 3, here too a range of motor performance and range of Y broader than provided by the DCD sample at hand is needed to answer the question. A broader range of motor proficiency in the sample, including cases with DCD according to the clinical criteria, would also shed light on whether the relationship is specific for DCD. However, we realize that for screened samples, it is not always possible to comply with the full set of clinical criteria and international and national guidelines to select the target group. In these cases, we propose to use the fifth percentile as a cutoff for the motor test, which reduces the chance of including false positives and increases the effect size (see also the discussion in [1]).

## General Conclusions

Consensus-based clinical diagnostic criteria as published in diagnostic manuals such as DSM are necessary to diagnose children with poor motor development who need intervention. Likewise, consensus is needed for research diagnostic criteria. Research diagnostic criteria serve the purpose to select children with DCD to investigate specific questions about the condition DCD, whether these are theoretical or practical in nature and whether these concern differences between DCD and typically developing children. Around the year 2000, the average number of articles published on DCD was 10, which increased to 60 per year around 2012 [9], most of these presenting original research. Consensus about and clear report of research diagnostic criteria will help to compare and integrate the results from these studies into understanding DCD.

In the present article, we argue that for research questions that focus on clinical aspects, DCD subjects should be selected that fully comply with the clinical diagnostic criteria. For specific research questions that study relationships between predictors and characteristics of DCD a continuum approach is more optimal than a group differences approach; here, preferably one should study the full range of the variables studied to understand the relationship and draw conclusions for the DCD range relative to the whole range. Typically, one would include a group of children with poor motor development, or even a clinical DCD group to fill in the lower range of the target motor variable. For research that selects target subjects by screening procedures, we prefer the use of the clinical criteria; however, if not all criteria can be thoroughly investigated, we propose a fifth percentile cutoff on the motor test. Finally, research is needed that compares clinical groups and groups selected for research purposes to know if knowledge gained in one group can be generalized to the other.

## Footnotes

1 Whenever we mention at or below 15th percentile, it could also be read as 16th, if this cutoff value is not available in the norms, like in the Movement ABC-2.
